# The Use of Bovine Pericardial Buttress on Linear Stapler Fails to Reduce Pancreatic Fistula Incidence in a Porcine Pancreatic Transection Model

**DOI:** 10.1155/2011/624060

**Published:** 2011-10-25

**Authors:** A. Maciver, M. McCall, D. Mihalicz, D. Al-Adra, R. Pawlick, A. M. J. Shapiro

**Affiliations:** Department of Surgery, University of Alberta Hospitals, 2000 College Plaza, 8215-112 Street, Edmonton, Alberta, Canada T6G 2C8

## Abstract

We investigate the effectiveness of buttressing the surgical stapler to reduce postoperative pancreatic fistulae in a porcine model. As a pilot study, pigs (*n* = 6) underwent laparoscopic distal pancreatectomy using a standard stapler. Daily drain output and lipase were measured postoperative day 5 and 14. In a second study, pancreatic transection was performed to occlude the proximal and distal duct at the pancreatic neck using a standard stapler (*n* = 6), or stapler with bovine pericardial strip buttress (*n* = 6). *Results*. In pilot study, 3/6 animals had drain lipase greater than 3x serum on day 14. In the second series, drain volumes were not significantly different between buttressed and control groups on day 5 (55.3 ± 31.6 and 29.3 ± 14.2 cc, resp.), nor on day 14 (9.5 ± 4.2 cc and 2.5 ± 0.8 cc, resp., *P* = 0.13). Drain lipase was not statistically significant on day 5 (3,166 ± 1,433 and 6,063 ± 1,872 U/L, resp., *P* = 0.25) or day 14 (924 ± 541 and 360 ± 250 U/L). By definition, 3/6 developed pancreatic fistula; only one (control) demonstrating a contained collection arising from the staple line. *Conclusion*. Buttressed stapler failed to protect against pancreatic fistula in this rigorous surgical model.

## 1. Introduction

The development of a postoperative pancreatic fistula (POPF) is a serious potential complication of any pancreatic surgery. Estimates of incidence vary by procedure and the underlying pancreatic pathology; however, when they occur, they can cause additional morbidity and occasional mortality [1]. A grading system for POPF has been developed by the International Study Group on Pancreatic Fistula Definition (ISGPF), and it provides a uniform terminology for assessment of clinical impact [2]. Generally, a POPF is defined as an abnormal communication between the pancreatic ductal epithelium and another epithelial surface, resulting in the leaking of fluid rich in pancreatic enzymes. 

Buttressing of the stapler to reinforce the staple line has received little attention in the setting of pancreatic surgery to date; however, this technique is gaining support and often used in other applications such as bariatric procedures [3, 4]. Of the few published reports, a bioabsorbable mesh buttress used in conjunction with standard stapler significantly reduced pancreatic leak rate [5, 6]. To date, there has yet to be a randomized controlled trial to investigate the effect that staple line reinforcement has on POPF. Our study is a preclinical evaluation of one such product in a porcine model of distal pancreatectomy to determine whether this confers added protection against POPF.

## 2. Methods

We herein investigate the potential beneficial role of a buttressed stapled pancreatic transection compared with nonbuttressed stapled pancreatic transection in a large animal (pig) clinically relevant surgical model of pancreatic surgical transection. We first evaluated the risk of pancreatic fistula in a straightforward distal pancreatectomy laparoscopic model, but found that this model had a relatively low rate of fistula formation. To avoid the need for large numbers of pigs and improve the sensitivity of the model, we developed a pancreatic transection model at the pancreatic neck, which left part of the pancreatic duct completely occluded distally, with the duodenal pancreatic lobe intact. While this is a perhaps a more stringent model compared to a typical clinical pancreatic transection in patients, we anticipated that this approach would more rigorously evaluate the strength of the pancreatic transection staple line with and without buttress.

## 3. Animals

Adult female swine were obtained from the colony at the Swine Research and Technology Centre, University of Alberta, Edmonton. Ethical approval was obtained from the University animal welfare committee and all animals were maintained according to the guidelines of the Canadian Council on Animal Care. Animals were fasted more than 12 hours before surgery.

### 3.1. Hand-Assisted Laparoscopic Distal Pancreatectomy

Animals underwent general anesthesia and intubation, and a laparoscopic approach was taken. A right lower transverse incision was made and a hand port was inserted (ETHICON, Somerville, NJ, USA) Three additional ports were placed in the left lower quadrant. The splenic lobe of the pancreas was identified, elevated, and freed from peritoneal attachments using the harmonic scalpel (ETHICON, Somerville, NJ, USA). The laparoscopic stapler (Echelon Flex, 45 mm, ETHICON, Somerville, NJ, USA) with a green cartridge was introduced through the 12 mm port. The gland was slowly closed between the arms of the stapler over 45 seconds, and a further 30 seconds allowed before the stapler was fired and cut. Approximately 4 cm of the distal gland was transected and removed via the hand port. A 14 F blake drain (ETHICON, Somerville, NJ, USA) was trimmed to 6 cm of functional drain and laid in the vicinity of the pancreatic bed. The drain was brought out laterally and tunnelled in the subcutaneous tissue to exit near the centre of the animal's back. Bulb suction was attached to the drain and placed in a pouch which was secured to the skin using adhesive tapes. The hand port incisions were closed by suturing the fascial layers, and skin incisions were reapproximated with staples. The animal was allowed to recover from the anaesthetic and given routine postop care including feed and water ad libitum. 

### 3.2. Pancreatic Transection at the Pancreatic Neck

**Figure 1:**
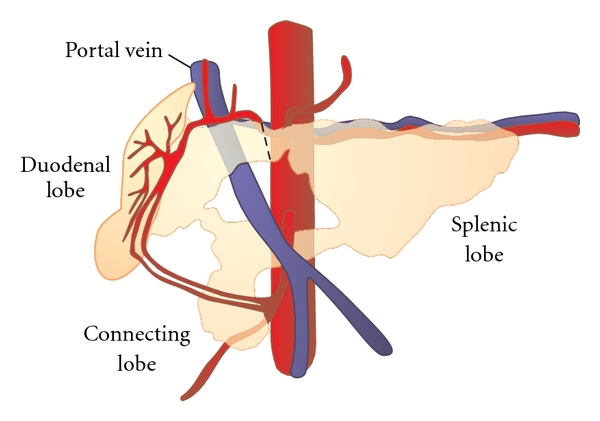
Anatomy of porcine pancreas. Dashed line indicates point of transection of gland between duodenal and splenic lobes, to the immediate left of the underlying portal vein.

Animals underwent general anaesthesia and intubation and a midline laparotomy was performed. Based on published data on anatomical drainage patterns of the porcine pancreas [7], the portion of gland between the duodenal and splenic lobes of the pancreas was chosen as the relevant point of duct occlusion (Figure 1). These two lobes were identified, and dissection of the gland to the left of and anterior to the portal vein was performed using blunt dissection and electrocautery. A plane was thus developed between the pancreas and the vein, and the laparoscopic stapler was introduced through this window. Animals were randomized to receive either standard 45 mm staple line, or staple line with a buttress of bovine pericardium (Peri-Strips Dry With Veritas Collagen Matrix Staple Line Reinforcement, Synovis Life Technologies, Minneapolis, Minn, USA). The stapler was fired as described above in the control group, and in the buttress group, the buttress was applied to the stapler before use as recommended by manufacturer. A 14 F Blake drain was trimmed to 6 cm functional length, laid along the transected ends of the gland and tacked loosely in place at two points using 3–0 PDS suture (ETHICON, Somerville, NJ, USA). The drain was brought out laterally and tunnelled in the subcutaneous tissue to the back as in the laparoscopic treatment group. The laparotomy incision was closed using a running suture of 1 PDS (ETHICON, Somerville, NJ, USA) and the skin was reapproximated with skin staples. The animal was allowed to recover from the anaesthetic and given routine postop care including feed and water ad libitum.

All operations were performed by one of two surgeons (A. M. J. Shapiro and A. Maciver).

## 4. Postoperative Course

All drains were placed to bulb suction and reprimed as needed, at least once daily. Drain output volumes were emptied and measured daily, and samples were taken for amylase and lipase on postoperative days 5 and 14. Jugular venous blood samples were also taken on these days for serum amylase and lipase. Pancreatic fistula was defined as a pancreatic fluid discharge for more than 14 days after operation diagnosed according to enzyme concentration in the drain fluid (lipase 3× greater than serum).

## 5. Examination on Necropsy

Following jugular venous and drain sampling on postoperative day 14, animals were euthanized, and necropsy was performed. Observations were made on the following: location and description of adhesions, intra-abdominal fluid collections, gross evidence of pancreatitis, staple line appearance, signs of infection, and signs of haemorrhage. Any fluid collections identified were sampled and sent for quantitative analysis of amylase and lipase. The pancreas was dissected and examined carefully for evidence of staple line disruption. 

## 6. Statistical Analysis

Statistical analysis was carried out using GraphPad Prism (Version 5.0b, GraphPad Software Inc., San Diego, Calif, USA). P values less than 0.05 were considered statistically significant. Graphical representation of data is represented as mean ± SEM. Means of drain and serum lipase were compared using unpaired *t*-tests. For values which were < or > than a laboratory range, the lower and upper limits were used for calculation, respectively. 

## 7. Results

**Figure 2:**
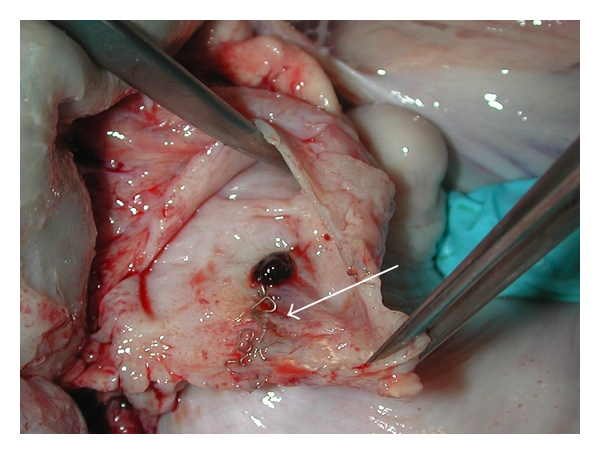
Necropsy photo of contained fistula arising from staple line (arrow) of distal pancreatectomy in one pig.

All animals in the laparoscopic hand-assisted distal pancreatectomy study recovered well from their procedure. Average drain volume on POD 5 was 3.3 ± 2.1 cc. One pig displaced its drain inadvertently on POD 10. Of the five remaining animals with drains, average drain volume on POD 14 was 7.2 ± 3.9 cc. Using a definition of drain fluid of any amount containing >3× serum level of lipase, 4/6 animals fulfilled the criteria for POPF at POD 14; however, only one had a demonstrated disruption of the staple line and contained fistula arising from it on necropsy (Figure 2). This same animal also had intra-abdominal fluid collections high in lipase. Results are summarized in Table 1.

**Figure 3:**
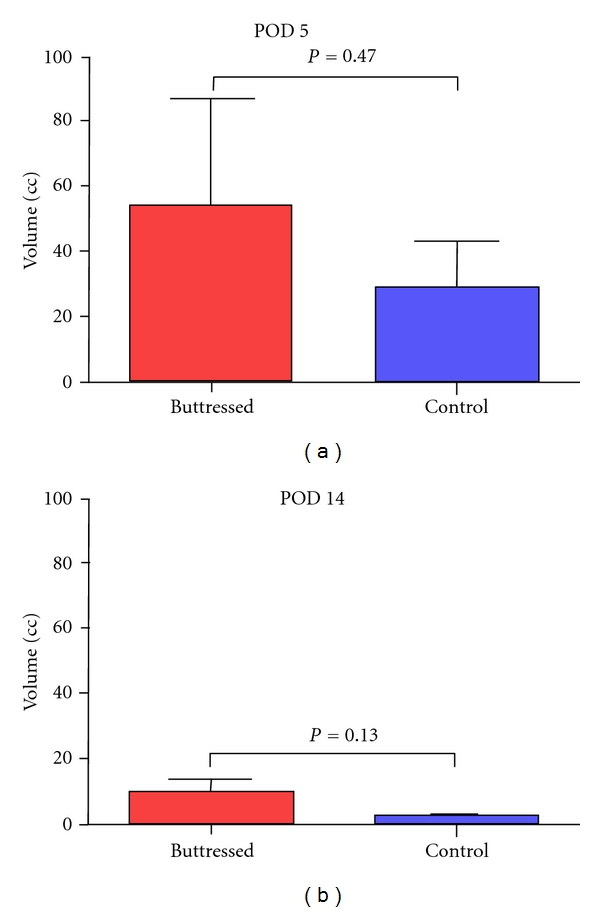
Drain volumes measured POD 5 and POD 14 comparing control and buttressed groups. *P* = NS.

**Figure 4:**
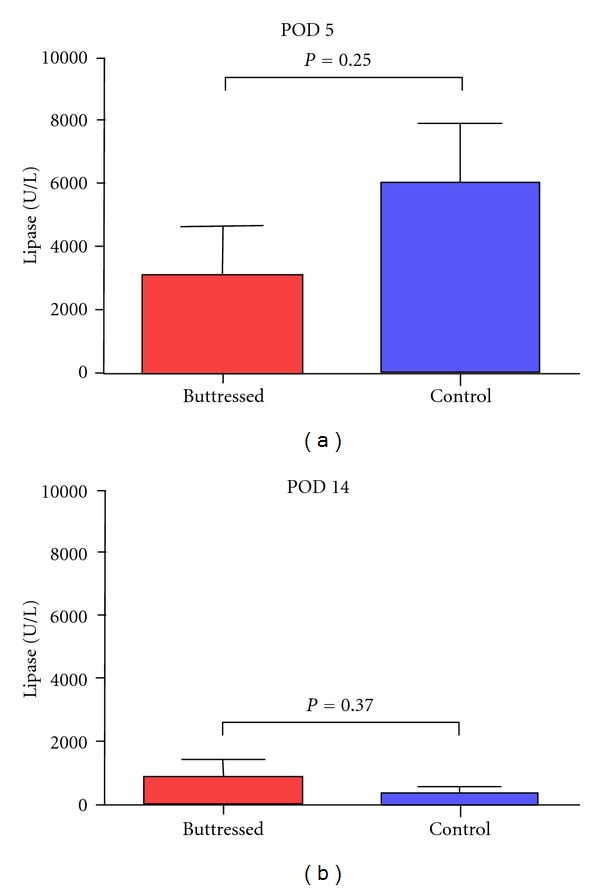
Lipase values in drain fluid POD 5 and POD 14 comparing control and buttressed groups (*P* = NS).

In the pancreatic transection model, POD 5 drain volumes were not significantly different between buttressed and control groups (55.3 ± 32.0 and 29.3 ± 14.2 cc, resp., *P* = 0.47), and while overall output declined by POD 14, there was no significant difference between the groups (9.5 ± 4.2 cc and 2.5 ± 0.8 cc, resp., *P* = 0.13) (Figure 3). Differences in drain fluid lipase were not statistically significant on POD 5 between control and buttressed techniques (3,166 ± 1,433 and 6,063 ± 1,872 U/L, resp.,  *P* = 0.25) or on POD 14 (924 ± 541  and 360 ± 250 U/L, *P* = 0.37) (Figure 4, data summarized in Table 2). By definition, 3/6 (50%) of control and 3/6 (50%) of buttressed animals had high concentrations of lipase (>3× serum) in the drain fluid. 

Three animals (two buttressed and one control) were found to have disruptions of the staple line on necropsy with evidence of an incorporated, contained fistula at the transection in one control animal. Of 12 animals, 10 had gross evidence of pancreatitis in the distal gland on necropsy.

## 8. Discussion

Distal pancreatectomy is indicated for neoplastic and benign lesions of the pancreatic body and tail and is a procedure increasingly performed laparoscopically [8]. Stapling devices are viewed as an efficient and safe tool to achieve a sealed pancreatic remnant, laparoscopically or open, though the evidence to date has not been uniformly in support of their use in all clinical situations. A multicentered, randomized, controlled, and patient- and observer-blinded trial is ongoing to compare conventional closure with stapling [9]. The burden of POPF in this setting represents a significant portion of the morbidity associated with the procedure, and despite the development of modifications in surgical technique to reduce this complication, it persists with an estimated incidence of 25%–30% [10–12]. Thus, as many surgeons elect to use a stapled technique, there has been interest in minimizing POPF by adding an adjunct to the device in the form of a bioabsorbable buttress [5, 6]. The buttress chosen is commercially available and has been used in other surgical applications; collagen matrix sourced from bovine pericardium is desirable for several reasons, including strength (collagen fibers are multidirectional) and low cellularity (biocompatible). 

Distal pancreatectomy is performed for a variety of indications, and on pancreatic tissue of varying texture and quality. Efforts have been made to determine the influence of different patient risk factors on the rate of POPF in this setting, but the incidence of POPF following this procedure is estimated to be in the range of 25%–30% [10–12]. A 2008 review suggested that data trends on fistula incidence, morbidity, and mortality rates have been stable since 1980 [13]; however, since that time, there have been many investigations comparing the efficacy of different pancreatic transection and stump closure techniques in an effort to reduce the frequency of this complication. 

To this end, studies have addressed direct treatment of the main pancreatic duct; main duct ligation, especially after suture closure of the stump, has been found to reduce POPF incidence [11, 14]. No improvement was noted, however, following the intracanal injection of fibrin sealant [15]. Other techniques have been examined as a device is given a novel application in distal pancreatectomy. For example, ultrasonic dissection in nonfibrotic pancreas (soft and without ductal dilatation) has been shown to be superior to scalpel division and suture closure in one randomized clinical trial [16], and transection using Ligasure is as effective as scalpel and suture closure in porcine models [17, 18]. Others have successfully reduced POPF incidence with the use of bipolar scissors to transect pancreas compared to conventional methods [19], and still another retrospective review suggests electrocautery with oversewing has the lowest rates of complication compared to scalpel division or stapling [20]. The pancreatic stump itself has been protected by a falciform pedicle flap [21], the round ligament of the liver plus fibrin glue [22], and fixation of the omentum with fibrin glue [23], all associated with reductions in POPF morbidity.

While stapling devices become increasingly commonplace in operating rooms, there appears to be no consensus regarding their impact on POPF reduction when compared to conventional methods of transection and even conflicting evidence in the literature. For this reason, the DISPACT study, a multicentered, randomized, controlled, and patient- and observer-blinded trial is ongoing to determine the efficacy of stapler versus hand-sewn closure of the pancreatic remnant [9]. 

The present study investigates the incidence of POPF in a large animal, stapled distal pancreatectomy model, and also the safety and efficacy of a bioabsorbable bovine pericardial buttress on a linear stapler in a preclinical large animal model of pancreatic transection. In the second model, the pancreas was divided and the duct occluded such that drainage of the splenic lobe of the porcine pancreas would be impeded, placing increased stress on the staple line. In 10/12 animals, there was evidence of pancreatitis in the distal portion but not the proximal portion of the gland. 

Serum and drain fluid that was collected for analysis was uniformly very high in amylase in nearly all animals (>2400 U/L), and lipase was chosen as an acceptable surrogate for evidence of pancreatic leak. 

Therefore, we find in the present study that the addition of a bioabsorbable buttress to the staple line fails to mitigate the risk of pancreatic fistula in this stringent model. Reasons for this may be related to pancreas tissue or the device itself. We recognize the major shortcoming of this study as the presence of a completely occluded pancreatic remnant staple line, which would not be tolerated in the clinical setting. We justified this approach based on a desire to attain sufficient sensitivity of the model without requiring a large cohort of pigs, which would have been prohibitive from a study cost perspective. Thus, we cannot exclude the possibility that a buttressed staple line in the absence of distal ductal obstruction might be beneficial in the clinical setting, but the strength of the buttressed staple line appeared to be inadequate to overcome fistula formation in 3 of 6 cases. We acknowledge that limited numbers per group restricts our ability to expand the analysis to a larger subset. The findings therefore are regarded as suggestive but not definitive.

## 9. Conclusions

In conclusion, this study indicates that buttressing of the staple line with a bioabsorbable buttress material in the setting of distal pancreatectomy is a potentially safe procedure. However, whether it confers additional protection against the morbidity of POPF remains to be proven, as it failed to reduce the incidence of this complication in our rigorous porcine transection ductal occlusion model compared to conventional stapling. Further investigation is warranted, as its use in different pancreatic texture and thicknesses may distinguish it as a worthwhile adjunct for protection of the pancreatic remnant.

## Figures and Tables

**Table 1:** Distal pancreatectomy with standard vascular stapler.

Pig	Drain volume (cc)		Drain lipase (U/L)		Serum lipase (U/L)		Staple line intact
	POD 5	POD 14	POD 5	POD 14	POD 5	POD 14	
1	1	n/a*	12	n/a*	<10	12	yes
2	1	0	80	39	14	10	yes
3	<1	22	32	13	10	<10	yes
4	2	8	60	32	133	<10	yes
5	14	4	83	>10000	82	10	no
6	<1	2	<10	50	17	<10	yes

*Drain misplaced POD 10.

**Table 2:** Comparison of control and buttressed animals.

Pig	Experimental group	Drain volume (cc)		Fluid lipase (U/L)		Serum lipase (U/L)		Staple line grossly intact	Fistula at end point?
		POD 5	POD 14	POD 5	POD 14	POD 5	POD14		
3	control	35	6	>10000	213	11	<10	yes	yes
4	control	18	<1	4831	1584	83	<10	no	yes
5	control	5	<1	1536	14	19	<10	yes	no
7	control	96	3	>10000	20	196	18	yes	no
10	control	20	2	>10000	318	48	10	no	yes
12	control	2	2	11	11	3210	11	yes	no
	Mean	29	3	6063	360	595	12	4/6 intact	3/6 fistula

1	buttressed	160	6	2673	799	24	<10	yes	yes
2	buttressed	4	29	199	10	<10	<10	yes	no
6	buttressed	150	9	>10000	1308	1369	46	no	yes
8	buttressed	10	10	3222	3401	13	32	yes	yes
9	buttressed	6	<1	1459	<10	14	<10	yes	no
11	buttressed	2	2	1441	16	11	10	yes	no
	Mean	55	10	3166	924	240	20	5/6 intact	3/6 fistula

## References

[1] Pedrazzoli S, Liessi G, Pasquali C, Ragazzi R, Berselli M, Sperti C (2009). Postoperative pancreatic fistulas: preventing severe complications and reducing reoperation and mortality rate. *Annals of Surgery*.

[2] Bassi C, Dervenis C, Butturini G (2005). Postoperative pancreatic fistula: an international study group (ISGPF) definition. *Surgery*.

[3] Alley JB, Fenton SJ, Harnisch MC, Angeletti MN, Peterson RM (2010). Integrated bioabsorbable tissue reinforcement in laparoscopic sleeve gastrectomy. *Obesity Surgery*.

[4] Shikora SA (2004). The use of staple-line reinforcement during laparoscopic gastric bypass. *Obesity Surgery*.

[5] Thaker RI, Matthews BD, Linehan DC, Strasberg SM, Eagon JC, Hawkins WG (2007). Absorbable mesh reinforcement of a stapled pancreatic transection line reduces the leak rate with distal pancreatectomy. *Journal of Gastrointestinal Surgery*.

[6] Jimenez RE, Mavanur A, MacAulay WP (2007). Staple line reinforcement reduces postoperative pancreatic stump leak after distal pancreatectomy. *Journal of Gastrointestinal Surgery*.

[7] Ferrer J, Scott WE, Weegman BP (2008). Pig pancreas anatomy: implications for pancreas procurement, preservation, and islet isolation. *Transplantation*.

[8] Casadei R, Ricci C, D’Ambra M (2010). Laparoscopic versus open distal pancreatectomy in pancreatic tumours: a case-control study. *Updates in Surgery*.

[9] Diener MK, Knaebel HP, Witte ST (2008). DISPACT trial: a randomized controlled trial to compare two different surgical techniques of DIStal PAnCreaTectomy—study rationale and design. *Clinical Trials*.

[10] Kah Heng CA, Salleh I, San TS, Ying F, Su-Ming T (2010). Pancreatic fistula after distal pancreatectomy: incidence, risk factors and management. *ANZ Journal of Surgery*.

[11] Goh BK, Tan YM, Chung YFA (2008). Critical appraisal of 232 consecutive distal pancreatectomies with emphasis on risk factors, outcome, and management of the postoperative pancreatic fistula: a 21-year experience at a single institution. *Archives of Surgery*.

[12] Weber SM, Cho CS, Merchant N (2009). Laparoscopic left pancreatectomy: complication risk score correlates with morbidity and risk for pancreatic fistula. *Annals of Surgical Oncology*.

[13] Parr ZE, Sutherland FR, Bathe OF, Dixon E (2008). Pancreatic fistulae: are we making progress?. *Journal of Hepato-Biliary-Pancreatic Surgery*.

[14] Bilimoria MM, Cormier JN, Mun Y, Lee JE, Evans DB, Pisters PWT (2003). Pancreatic leak after left pancreatectomy is reduced following main pancreatic duct ligation. *The British Journal of Surgery*.

[15] Suc B, Msika S, Fingerhut A (2003). Temporary fibrin glue occlusion of the main pancreatic duct in the prevention of intra-abdominal complications after pancreatic resection: prospective randomized trial. *Annals of Surgery*.

[16] Suzuki Y, Fujino Y, Tanioka Y (1999). Randomized clinical trial of ultrasonic dissector or conventional division in distal pancreatectomy for non-fibrotic pancreas. *The British Journal of Surgery*.

[17] Hartwig W, Duckheim M, Strobel O (2010). Liga sure for pancreatic sealing during distal pancreatectomy. *World Journal of Surgery*.

[18] Gehrig T, Fonouni H, Mueller-Stich BP Comparison of different surgical techniques in distal pancreatectomy: an experimental study in a porcine model.

[19] Kawai M, Tani M, Yamaue H (2008). Transection using bipolar scissors reduces pancreatic fistula after distal pancreatectomy. *Journal of Hepato-Biliary-Pancreatic Surgery*.

[20] Harris LJ, Abdollahi H, Newhook T (2010). Optimal technical management of stump closure following distal pancreatectomy: a retrospective review of 215 cases. *Journal of Gastrointestinal Surgery*.

[21] Walters DM, Stokes JB, Adams RB, Bauer TW (2011). Use of a falciform ligament pedicle flap to decrease pancreatic fistula after distal pancreatectomy. *Pancreas*.

[22] Iannitti DA, Coburn NG, Somberg J, Ryder BA, Monchik J, Cioffi WG (2006). Use of the round ligament of the liver to decrease pancreatic fistulas: a novel technique. *Journal of the American College of Surgeons*.

[23] Velanovich V (2007). The use of tissue sealant to prevent fistula formation after laparoscopic distal pancreatectomy. *Surgical Endoscopy and Other Interventional Techniques*.

